# Do Unto Others: Doctors' Personal End-of-Life Resuscitation Preferences and Their Attitudes toward Advance Directives

**DOI:** 10.1371/journal.pone.0098246

**Published:** 2014-05-28

**Authors:** Vyjeyanthi S. Periyakoil, Eric Neri, Ann Fong, Helena Kraemer

**Affiliations:** 1 Stanford University School of Medicine, Palo Alto, California, United States of America; 2 Veterans Affairs (VA) Palo Alto Health Care System, Palo Alto, California, United States of America; 3 Stanford Hospital and Clinics, Palo Alto, California, United States of America; The James Cook University Hospital, United Kingdom

## Abstract

**Objective:**

High-intensity interventions are provided to seriously-ill patients in the last months of life by medical sub-specialists. This study was undertaken to determine if doctors' age, ethnicity, medical sub-specialty and personal resuscitation and organ donation preferences influenced their attitudes toward Advance Directives (AD) and to compare a cohort of 2013 doctors to a 1989 (one year before the Patient Self Determination Act in 1990) cohort to determine any changes in attitudes towards AD in the past 23 years.

**Design:**

Doctors in two academic medical centers participated in an AD simulation and attitudes survey in 2013 and their responses were compared to a cohort of doctors in 1989.

**Outcomes:**

Resuscitation and organ donation preferences (2013 cohort) and attitudes toward AD (1989 and 2013 cohorts).

**Results:**

In 2013, 1081 (94.2%) doctors of the 1147 approached participated. Compared to 1989, 2013 cohort did not feel that widespread acceptance of AD would result in less aggressive treatment even of patients who do not have an AD (p<0.001, AUC = 0.77); had greater confidence in their treatment decisions if guided by an AD (p<.001, AUC = 0.58) and were less worried about legal consequences of limiting treatment when following an AD (p<.001, AUC  = 0.57). The gender (p = 0.00172), ethnicity (χ^2^ 14.68, DF = 3,p = .0021) and sub-specialty (χ^2^ 28.92, p = .004, DF = 12) influenced their attitudes towards AD. 88.3% doctors chose do-not-resuscitate status and wanted to become organ donors. Those less supportive of AD were more likely to opt for “full code” even if terminally ill and were less supportive of organ donation.

**Conclusions:**

Doctors' attitudes towards AD has not changed significantly in the past 23 years. Doctors' gender, ethnicity and sub-specialty influence their attitudes towards AD. Our study raises questions about why doctors continue to provide high-intensity care for terminally ill patients but personally forego such care for themselves at the end of life.

## Introduction

The silver tsunami of older adults is perhaps the largest public health challenge facing society today. Advances in modern biomedicine have resulted in unprecedented increases in longevity and to some extent in compression of morbidity. However, they have failed to significantly improve health status in the last two years just prior to death resulting in millions of Americans living with the tremendous burden of major chronic disease(s) at the end of life [1]. In 2005, 133 million Americans (almost 50% of the adult population) had at least one chronic illness [2]. Seven out of ten deaths each year are from chronic diseases with heart disease, cancer and stroke accounting for more than 50% of all deaths [3]. Older Americans account for an estimated 32% of the total Medicare spending on costs related to repeated hospitalizations in the last two years of their life and higher spending has not been associated with better health outcomes [4]. A recent Dartmouth Atlas report [5] showed that end-of-life care in the US is more fragmented than ever before. Nationally, there was a twelve percentage point increase in Medicare beneficiaries who saw more than ten different physicians in the last six months of life, especially medical subspecialists, and spent more days in intensive care units in 2010 compared to 2003-07

A big disparity exists [6] between what Americans say they want at the end-of-life (EOL) and the care they actually receive. More than 80% of patients say [7] that they wish to avoid hospitalizations and high intensity care at the end-of-life, but their wishes are often overridden. Most patients at the end-of-life prefer care that is focused on augmenting their comfort and dignity [8]–[9] and wish to die a gentle and natural death at home without burdening their families financially or emotionally [4]–[6].The current gap between the care Americans want and what they receive at the end-of-life is not likely due to patient and family choice [10]–[11] nor do differences in patients' preferences *per se* explain regional variations [4] seen nationally in EOL spending. Studies show that the end-of-life care patients receive depends not on the patients' care preferences or their advance directives (AD) but rather on the local health care system variables like institutional capacity and individual doctors' practice style [5], [10]. These two variables also explain [10], [12] the tremendous regional variation in Medicare spending on patients at the end-of-life.

While doctors may favor Advance Directives (AD) in theory, they favor them less compared to their patients [13] and use them infrequently [14]. Also, when the patients' documented AD wishes [14]–[16] are in conflict with the doctor's clinical opinion about what is best the patient, doctors may override [7] patient autonomy in favor of doing what they (the doctors) perceive as beneficial to the patient [17]. In order for patients to consistently receive preference-sensitive care, in addition to the patient documenting AD, their doctors must be willing to honor, implement and facilitate the patient's advance directives and their wishes for care at the end of life.

As an increasing number of sub-specialists are providing care to patients in the last six months of life [4], the current study was undertaken to test if (a) doctors' age, ethnicity, medical sub-specialty and a doctor's personal resuscitation and organ donation preferences influence their attitudes towards Advance Directives (AD) and (b) to compare the responses of a 2013 cohort of doctors to a 1989 (one year before the passage of the Patient Self Determination Act in 1990) cohort of doctors to determine any changes in attitudes towards advance directives in the past 23 years.

## Methods

### Study Population and Data

Medical sub-specialists who care for seriously ill patients participated at the end of the clinical training year just before graduation. Data were collected as a part of a quality improvement project during March through July 2013 with the participants' knowledge. The Stanford institutional review board approval was obtained to analyze the data presented in this paper. Of 1147 potential participants, 1081 participated (94.2 % response rate). Participants completed a web-based AD form and a 14 item AD attitude survey. The measures were administered online and required no Personal Health Identifiers in an effort to promote participant confidentiality and honest responses without concerns about individual scrutiny. Study sites included Stanford Hospital and Clinics and the VA Palo Alto Health Care System, two large training hospitals in California.

### Outcome measures

1. **a. Doctors' attitudes towards advance directives.** In 1989, the Journal of the American Medical Association published a study [18] examining physicians' opinions of the standard arguments for and against advance directives. In this paper Davidson *et al* [18] surveyed 790 physicians in practice in Arkansas using a 14 item AD attitudes questionnaire to assess doctors opinions towards advance directives. We used the same 14 item questionnaire and compared responses of 790 doctors from the 1989 cohort (control group) to a 2013 cohort of doctors in two large academic hospitals. Our goal was to better understand the current attitudes of doctors and to assess for any changes in doctors' attitudes toward advance directives (AD) over the last 23 years since the passage of the Patient Self Determination Act (PSDA) in 1990 [19]. We hypothesised that as advance directives documentation has become a routine and accepted healthcare practice, current day doctors attitudes would be much more positive towards AD compared to the control group in 1989 (an year before the PSDA was passed).
2. **b. 2013 participants resuscitation preferences.** All participants in the 2013 cohort completed a simulated California state advance directives document in which they indicated their end of life choices:
  1. **Code status or Resuscitation preferences**: Participants indicated their end of life code status preference as either
    1. ***Choice To Prolong (full-code status)***: “I want my life to be prolonged as long as possible within the limits of generally accepted medical treatment standards” (or)
    2. ***Choice Not To Prolong (no-code)***: “I do not want my life to be prolonged if the likely risks and burdens of treatment would outweigh the expected benefits, or if I become unconscious and, to a realistic degree of medical certainty, I will not regain consciousness, or if I have an incurable and irreversible condition that will result in my death”.
  2. **Opinions about organ donation**: whether or not participants were willing to become organ donors.

Sociodemographic characteristics included specialty, age group, gender, ethnicity/race were also collected for the 2013 cohort.

### Statistical Analysis

**Figure 1:**
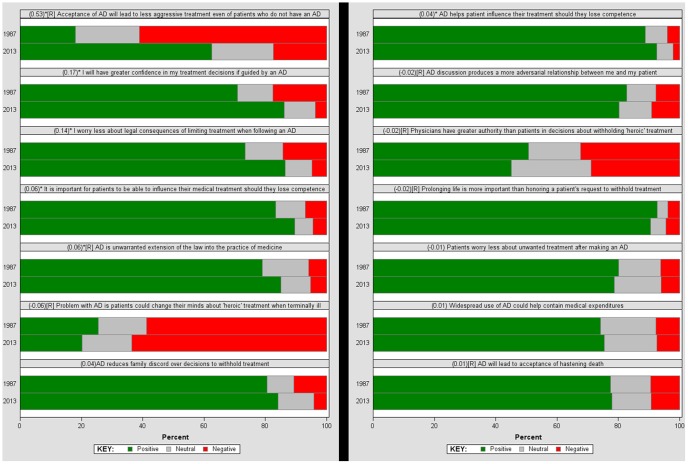
Comparison of the 1989 cohort to the 2013 cohort of doctors on each of the 14 statements about Advance Directives. Please note that ( ) indicates the Success Rate Difference Effect Size (−1.0 to +1.0). [R] indicates statements that oppose Advanced Directives. An asterisk sign* indicates the Mann-Whitney Wilcoxon test with the Bonferroni adjustment is significant at α = 0.05/14.

Analyses were done using the SAS software (SAS 9.3, SAS Inc., North Carolina). Of the 1147 potential participants in the 2013 cohort, fourteen did not respond. Fifty two participants who were undecided on all 14 survey items in the advance directives attitudes survey were not used in analyses. Participant demographics of both the 1989 and the 2013 cohort are shown in Table 1. The final 2013 cohort included n = 1081 doctors (94.2% response rate) and their responses on the 14 survey items were compared to those of the 1989 cohort [18] (see Figure 1). For each of the 14 survey items, responses in support of AD are shown in green and those in opposition of AD in red and those who were neutral are shown in gray. The area under the receiver operating characteristic curve (AUC) and the Success Rate Difference (SRD) [20]–[21] was calculated for each of the 14 items for both the 1989 and 2013 cohorts and are shown reordered based on the magnitude of the SRD (which measures the rate difference of a specific outcome) ranging from (−1) to (+1) with zero indicating no difference. Results for the 14 items were calculated significant at α = .05/14 with the Bonferroni adjustment and shown reordered by SRD, differentiating the 1989 and 2013 cohorts.

**Table 1:** Comparative demographics of participants.

CHARACTERSTIC	1989 PARTICIPANTS		2013 PARTICIPANTS	
	N	N	N	%
GENDER				
Female	57	7.5%	556	51.4
Male	700	92.5%	525	48.6
AGE				
<30 years	NA	NA	425	39.3
30–39 years	NA	NA	631	58.4
>40 years	NA	NA	25	2.3
RACE/ETHNICITY				
White or Caucasian	NA	NA	552	51.1
Hispanic or Latino American	NA	NA	57	5.3
African American	NA	NA	35	3.2
Asian American	NA	NA	353	32.7
Other	NA	NA	84	7.8
SUB-SPECIALTY				
Anesthesiology	0	0%	117	10.8
Emergency Medicine	0	0%	60	5.6
Family & Internal Medicine	757	100%	259	24.0
Neurology	0	0%	43	4.0
Obstetrics & Gynecology	0	0%	24	2.2
Orthopedics	0	0%	26	2.4
Pathology	0	0%	56	5.2
Pediatrics	0	0%	144	13.3
Physical Medicine & Rehabilitation	0	0%	32	3.0
Psychiatry	0	0%	59	5.5
Radiology & Nuclear Medicine	0	0%	82	7.6
Radiation Oncology	0	0%	22	2.0
Surgery	0	0%	157	14.5

### Calculation of the Average Advance Directive Supportive Score (AADSS)

For each of the 14 items, the 2013 participant responses were recoded as follows: strongly disagree (−1), disagree (−0.5), undecided (0), agree (+0.5) and strongly agree (+1.0). Next, for each participant, the scores on all the 14 items were averaged to obtain the individual's composite Average Advance Directive Support Score (AADSS). The Mann-Whitney-Wilcoxon test [22]–[23] was used to do subgroup analysis of the AADSS of each participant by gender and the Kruskal-Wallis [24] test was used to do sub-group analyses by age group, ethnicity/race and medical sub-specialty. To note, the AADDS scoring system could not be used for the 1989 cohort as the Likert scores in that study were collapsed from a 5 point to a three point scale as shown in their publication [18].

Finally, the Receiver Operating Characteristics (ROC) [25] procedure for recursive partitioning was used to analyze the patterns between various individual predictor variables (in this case, participants' age, gender, ethnicity/race, sub-specialty and AADSS). The ROC procedure analyses all possible cutpoints and combinations, and identifies the variable and its cutpoint with the optimal balance between sensitivity and specificity for identifying those particular subjects with the specific outcome of interest. The total group is then divided into two subgroups—those above and below the selected cutpoint on the selected variable—and the process is reiterated until no further discrimination was achieved. Two separate ROC analyses were computed. The first ROC analyses was based on the participant resuscitation preferences in event of terminal illness (“prolong” versus “do not prolong”) and the second analyses was on participants who were interested in organ donation versus those who were not.

## Results

The 2013 sample is larger (n = 1081 in 2013 and n = 790 in 1989) and includes all subspecialists ranging from medicine, surgery, gynecology and obstetrics, emergency medicine etc. The 1989 cohort [18] included only Internal Medicine and Family Medicine specialists. Notably, the 2013 cohort has a higher percentage of women (51.4 % women in the 2013 cohort compared to 7.5% women in the 1989 cohort) and diverse doctors (48.9% ethnic minorities in 2013; ethnicity was not reported in the 1989 cohort). This likely reflects the increasing influx of women and minorities into medicine as represented in recent national data [26]. The median AADSS for the 2013 cohort was 0.46 (25^th^ percentile  = 0.32 and 75^th^ percentile  = 0.57) indicating that doctors were overall disposed positively towards AD. There was a small but significant difference by gender with women displaying more positive attitudes towards advance directives compared to male doctors. Median AADSS scores was 0.46 for women (25^th^ percentile  = 0.32, 75^th^ percentile  = 0.61) and 0.43 for men (25^th^ percentile  = 0.32, 75^th^ percentile  = 0.57), p = 0.00172, SRD = 0.110. There were no significant differences by age group. Median was 0.46 for the 20–29 years age group (25^th^ percentile  = 0.36, 75^th^ percentile  = 0.61) and 0.43 for 30–39 years age group (25^th^ percentile  = 0.32, 75^th^ percentile  = 0.57), p = 0.0314, and SRD  = 0.078.

There were significant differences between ethnic groups (Chi-Square  = 14.68, DF = 3 and p = .0021). The major differences were seen between Caucasian vs. Hispanic/Latino doctors (SRD  = 0.207) and African American vs. Hispanic/Latino doctors (SRD = 0.220). To note, there were no significant differences between the Caucasian and African American doctors with both groups being equally positive towards AD. The Caucasians and African Americans doctors were most positively oriented towards advance directives followed by the Asian doctors and the Hispanic/Latino doctors were least positively oriented towards advance directives (see table 2).

**Table 2:** Comparison of sub-groups in the 2013 cohort based of self-reported ethnicity/race by the Average Advance Directives Support Score (AADDS).

Ethnicity/Race	N	25^th^ percentile	50^th^ percentile	75^th^ percentile
Caucasian	552	0.36	0.46	0.61
African American	35	0.36	0.46	0.61
Asian	353	0.32	0.43	0.57
Hispanic/Latino	57	0.29	0.39	0.54

The higher the AADSS score, the more positive the attitude towards Advance Directives ( Range of −1 to +1).

**Figure 2:**
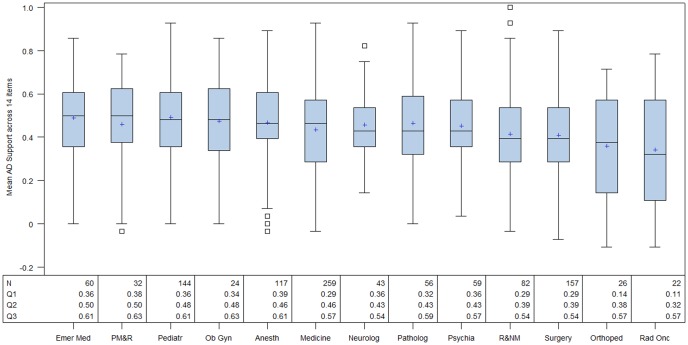
Box and Whisker plots of the 2013 participants sorted by subspecialty and ordered based on median Average Advance Directives Support Score (AADSS). The numbers in each sub-specialty, the 25th percentile (Q1), the median (Q2) and the 75th percentile (Q3) values are shown. The Emergency Medicine sub-specialists have the highest median AADSS score of 0.50 and Radiation Oncology sub-specialists are at a median AADDS of 0.32. Emer Med  =  Emergency Medicine, PM&R  =  Physical Medicine and Rehabilitation, Pediatr = Pediatrics, ObGyn =  Obstetrics and Gynecology, Anesth = Anesthesiology, Neurolog = Neorology, Pathlog = Pathology, Psychia = Psychiatry, R&NM =  Radiology and Nuclear Medicine, Orthoped =  Orthopedics, Rad Onc = Radiation Oncology. N = number of participants.

There were significant differences in attitudes towards advance directives by sub-specialty, (Chi Square = 28.92, p = .004, DF = 12). Doctors from Emergency Medicine, Physical Medicine and Rehabilitation, Pediatrics and Obstetrics and Gynecology were most positively disposed towards advance directives. Doctors specializing in Radiology and Nuclear Medicine, Surgery, Orthopedics and Radiation Oncology were least positively oriented towards advance directives (see Figure 2) The major differences were seen between Emergency Medicine vs. Radiation Oncology specialists (SRD  = 0.305); Pediatrics vs. Radiation Oncology specialists (SRD  =  0.304); Emergency Medicine vs. Orthopedics specialists (SRD  = 0.283); and Obstetrics and Gynecology vs. Radiation Oncology specialists (SRD  =  0.280).

In comparing the attitudes towards advance directives of the doctors in the 1989 cohort to those of the 2013 cohort, it is to be noted that there were statistically significant changes in the doctors attitudes in only three items (See table 3). Compared to 1989 cohort, the 2013 doctors (a) were unlikely to believe that AD will lead to less aggressive treatments even for patients who do not have an AD (p<.001, SRD = 0.53) (b) had greater confidence in their treatment decisions if guided by an AD (p<.001, SRD = 0.17) and (c) were less worried about legal consequences of limiting treatment when following an AD (p<.001, SRD = 0.14). There were no significant differences in the doctors' attitudes across the 1989 and 2013 cohorts in 11 out of the 14 items in the AD attitude survey (see figure 1) indicating perhaps that doctors' attitudes towards advance directives have not changed significantly in the past 23 years.

**Table 3:** Comparison of participants' responses on the advance directives attitudes questionnaire.

STATEMENTS	1989 PARTICIPANTS			2013 PARTICIPANTS					AUC	SRD
	Agree or Strongly Agree, %	Undecided %	Disagree or Strongly Disagree %	Strongly Agree %	Agree %	Undecided%	Disagree %	Strongly Disagree %		
**Widespread acceptance of advance directives will lead to less aggressive treatment even of patients who do not have an advance directive.**	**61.2**	**20.7**	**18.1**	**3.7**	**13.8**	**20.0**	**42.8**	**19.7**	**0.77**	**0.53**
				**17.5**		**20.0**	**62.5**			
**In a catastrophic situation, I would have greater confidence in my treatment decisions if guided by an advance directive.**	**70.9**	**11.5**	**17.6**	**28.9**	**57.3**	**10.2**	**3.4**	**0.3**	**0.58**	**0.17**
				**86.1**		**10.2**	**3.7**			
**I would worry less about legal consequences of limiting treatment if I were following an advance directive.**	**73.4**	**12.3**	**14.3**	**32.3**	**54.1**	**8.8**	**4.1**	**0.7**	**0.57**	**0.14**
				**86.4**		**8.8**	**4.8**			
**It is important for patients to be able to influence their medical treatment should they lose competence**	**83.3**	**9.8**	**6.9**	**40.5**	**49.0**	**6.0**	**3.4**	**1.0**	**0.53**	**0.06**
				**89.5**		**6.0**	**4.4**			
**Advance directives represent an unwarranted extension of the law into the practice of medicine**	**6**	**15**	**79**	**1.1**	**4.3**	**9.5**	**51.4**	**33.7**	**0.53**	**0.06**
				**5.4**		**9.5**	**85.1**			
**A potential problem with advance directives is that patients could change their minds about “heroic” treatment after becoming terminally ill.**	**58.8**	**15.7**	**25.4**	**8.3**	**55.3**	**16.1**	**16.7**	**3.5**	**0.47**	**−0.06**
				**63.6**		**16.1**	**20.3**			
**An advance directive would reduce family discord over decisions to withhold treatment.**	**80.6**	**8.6**	**10.8**	**28.1**	**56.1**	**11.7**	**3.7**	**0.5**	**0.52**	**0.04**
				**84.2**		**11.7**	**4.2**			
**Advance directives are an effective way for patients to influence their medical treatment should they lose competence.**	**88.6**	**7.2**	**4.1**	**34.9**	**57.7**	**5.3**	**1.7**	**0.5**	**0.52**	**0.04**
				**92.6**		**5.3**	**2.1**			
**Discussion of an advance directive would produce a more adversarial relationship between me and my patient.**	**7.8**	**9.5**	**82.6**	**1.7**	**7.5**	**10.5**	**53.6**	**26.7**	**0.49**	**−0.02**
				**9.2**		**10.5**	**80.3**			
**The training and experience of physicians gives them greater authority than patients in decisions about withholding “heroic” treatment.**	**32.4**	**16.9**	**50.7**	**4.9**	**24.1**	**26.0**	**34.5**	**10.5**	**0.49**	**−0.02**
				**29.0**		**26.0**	**45.1**			
**Prolonging life is more important than honoring a patient**'**s request to withhold “heroic” treatment.**	**3.9**	**3.4**	**92.7**	**1.0**	**3.4**	**5.1**	**29.3**	**61.1**	**0.49**	**−0.02**
				**4.4**		**5.1**	**90.5**			
**Patients would worry less about unwanted treatment after making an advance directive**	**80.1**	**13.7**	**6.2**	**17.9**	**60.9**	**15.2**	**5.5**	**0.6**	**0.49**	**−0.01**
				**78.7**		**15.2**	**6.1**			
**Widespread use of advance directives could help contain medical expenditures**	**74.1**	**18.1**	**7.7**	**23.4**	**52.0**	**17.1**	**5.2**	**2.3**	**0.51**	**0.01**
				**75.4**		**17.1**	**7.5**			
**I am concerned that advance directives will lead to acceptance of euthanasia.**	**9.5**	**13**	**77.5**	**1.2**	**8.1**	**12.7**	**50.3**	**27.7**	**0.5**	**0.01**
				**9.3**		**12.7**	**78.0**			

This table shows the responses of the 1989 cohort and the 2013 cohort on each of the items in the Advance Directives attudues assessment survey. AUC is the Area under the Reciever Operating Curve and SRD stands for Success Rate Difference.

### Receiver Operating Characteristics Analyses

The majority (n = 954 or 88.3%) of the 2013 doctors opted for the Do-Not-Prolong Life (no-code status) for themselves when terminally ill. Only 11.7% of the doctors opted for the Choice-to-Prolong Life (full-code status) for themselves. Doctors who were less supportive of AD were more likely to opt for full-code for themselves and were less likely to opt for organ donation. Of participants whose AADSS ≥0.21, 89.6% opted to be “no-code” compared to only 77.9% who opted for no-code if their AADSS <0.21. Of those doctors who were more supportive of AD (AADSS ≥0.21), 91.1% opted to become total or partial organ donors compared to only 76.8% in those whose AADSS <0.21.

## Discussion

Our data show that the doctors' attitudes' towards advance directives is remarkably similar in both the 1989 [18] and the 2013 cohorts. The 1989 cohort is a group of community doctors in practice in Arkansas and the 2013 is a younger and more diverse group of doctors in California from two academic hospitals. Our hypothesis that the passage of the PDSA 23 years ago and the subsequent broad acceptance of advance directives nationally would have resulted in a very positive attitude change in doctors towards advance directives was disproved. There were mostly no significant differences between the 1989 and 2013 cohorts responses to the AD attitude survey items (except for three items described above) leading us to infer that much work needs to be done to positively change doctors attitudes towards advance directives. As research has shown that individual doctors practice style is a more important variable then patients' own preferences in influencing the care they receive at the end of life, our study findings highlight the great need to implement national educational and policy changes to improve doctors' attitudes towards advance directives. Such efforts need to take into account the differences in attitudes towards advance directives seen across subspecialties. Our data show that within subspecialties, there were major differences in attitudes towards AD with doctors specialized in Emergency Medicine, PM&R, Pediatrics, Obstetrics & Gynecology and Anesthesia being most positively oriented compared to those in Surgery, Orthopedics and Radiation Oncology. It is possible that the surgical specialties are less positive towards AD as major surgical procedures often necessitate life support in the peri-operative and post-operative periods until the patient has recovered from the surgery and is stable enough to be discharged from the hospital. Data [27]–[30] also show that many surgeons do not routinely discuss AD preoperatively and decline to operate [27] on patients whose directives limit high-intensity care. Patients who are no-code at baseline are converted to full-code during the peri- and post- operative period [28]. It is important to revoke the temporary full-code status and readdress AD with patients at the time of discharge from the hospital after surgery.

Finally, our data show that doctors predominantly wish to forego high-intensity treatments for themselves at the end-of-life with 88.3% of our 2013 cohort opting to be no-code. Data from the Precursors study [31]–[32] showed that older physicians who had completed advance directives were more likely to opt for the “do not prolong” choice. To the best of our knowledge, our study is the first to analyze resuscitation preferences of a large and diverse cohort of younger doctors and to determine that they too predominantly opt for comfort care for themselves at the end of life.

Current national data [4] show very clearly that terminally ill Americans receive care from many sub-specialists in the last six months of life and are subjected to ineffective high-intensity treatments only to die expected deaths from known chronic illnesses. An important question our study raises is why doctors choose to forego high-intensity treatments for themselves at the end-of-life but continue to provide such care to their terminally ill patients? In other words, why are doctors choosing care for themselves that is very different from what they provide to their patients?

It is possible that the terminally ill patients are asking to be subjected to these high intensity treatments at the end of life. However, data [5]–[6] do not support this and in fact demonstrate that seriously ill patients prefer to die gently and naturally at home. The local health system culture and doctors' practice styles [5], [10] are the primary variables that result in high intensity treatments given to patients at the end of life. It is to be noted that, populations receiving higher care intensity in the last six months of life do not have lower mortality rates compared to populations receiving lower intensity care [4]. This then begs the question of what biases and incentives underlie the prevalent national practice pattern of subjecting dying patients to ineffective, burdensome high-intensity treatments though doctors choose low intensity EOL treatments for themselves. The reasons are likely multi-factorial and very complex.

Firstly, it is likely that doctors recurrently witness the tremendous suffering their terminally ill patients experience as they undergo ineffective, high intensity treatments at the end of life and they (the doctors) consequentially wish to forego such treatments for themselves. Second, doctors tend to be very optimistic [33]–[35] and overestimate the prognosis and life-span of their patients. This results in escalation of high burden technological interventions until it is clear to all stakeholders that the patient is dying. Sadly, this clarity often comes in the last few hours to days of life, resulting either in terminally ill patients experiencing highly medicalized death in hospitals or in very late referrals to hospice care. Accurate methods of prognostication will help both doctors and patients structure the care-plan based on more realistic estimates of patient's anticipated lifespan.

Thirdly, an important factor influencing the current state of healthcare is the culture of modern biomedicine with its default set to maximal interventions for all patients, irrespective of the effectiveness of doing so. This may foster implicit biases in doctors causing them to override their patients' autonomy when the patients' choices are in conflict with what the doctors believe will benefit the patient. While data [36] show that early palliative care is beneficial to patients and families, much work needs to be done to incorporate palliative care into the genome of modern biomedicine. Effort needs to be directed at creating a system infrastructure that automates the seamless and early integration of palliative care into the care of all patients with serious illness.

Finally, the current fiscal system rewards hospitals and doctors for medical procedures and providing high-intensity care to terminally ill persons and does not reimburse them for conducting prophylactic conversations that elicits values and goals of care and what matters most to patients and their families at the end of life. Most Americans are dying of chronic illnesses and currently a quarter of the total Medicare budget is spent on services to beneficiaries in the last year of life, with 40% of it on patients within the last 30 days of life [37]. Policy changes are required that promote, institutionalize and reward care practices that incorporate advance care planning and early palliative care for all seriously ill persons. A flexible range of options, tailored to the local institutional culture and the individual patient's preferences, should be available in the early provision of palliative care services.

There are limitations to the generalizability of this study. First, the 2013 study participants were doctors from two hospitals in one geographic area. However, recent national data [26], [38] from the Accreditation Council for Graduate Medical Education confirm an increasing influx of women and ethnic minorities comparable to our participants. 51.4% of our participants were women compared to the 2013 national data [38] of 46.1%. Race and ethnicity distribution of our cohort was White 51.1%, Asian 32.7%, Black 3.2% and Hispanic/Latino 5.3% compared to the 2013 national data of White 65.1%, Asian 21.2%, Black 6.3% and Hispanic/Latino 6.3%. Second, this was a cross-sectional study and it is possible that the participants' end-of-life wishes may change over time though data show [39] that preferences of doctors who chose lesser intensity care remain stable over time.

### Conclusions

Data [4] show that there is accelerating fragmentation of care of seriously ill Americans at the end-of-life. Dying patients continue to be hospitalized [40] and subjected to ineffective therapies that erode their quality of life and their personal dignity [8], [9]. Doctors' attitudes have hardly changed in the past 23 years despite the passage of the PSDA [19]. Our data show that doctors they have a striking personal preference to forego high-intensity care for themselves at the end-of-life and prefer to die gently and naturally. This study raises questions about why doctors provide care, to their patients, which is very different from what they choose for themselves and also what seriously ill patients want.

## References

[1] Cutler DM, Ghosh K, Landrum MB (2013) Evidence for significant compression of morbidity in the elderly US population; Working Paper 19268 National Bureau of Economic Research, 1050 Massachusetts Avenue, Cambridge, MA 02138.

[2] Wu SY, Green A (2000) Projection of chronic illness prevalence and cost inflation. Santa Monica, CA: RAND Health;.

[3] Kung HC, Hoyert DL, Xu JQ, Murphy SL (2008) Deaths: final data for 2005. National Vital Statistics Reports 2008;56(10). Available: http://www.cdc.gov/nchs/data/nvsr/nvsr56/nvsr56_10.pdf 18512336

[4] The Dartmouth Atlas of Health Care website: Available: http://www.dartmouthatlas.org/keyissues/issue.aspx?con=1338.Accessed 2013 Oct 1.

[5] The Dartmouth Atlas of Health Care website: Available: http://www.dartmouthatlas.org/downloads/reports/EOL_Trend_Report_0411.pdf Accessed 2013 Oct 1.

[6] The California Health Care Foundation website: Available http://www.chcf.org/media/press-releases/2012/end-of-life-care#ixzz2eLhnscM1 Accessed 2013 Oct 1.

[7] The Dartmouth Atlas of Health Care website: Available http://www.dartmouthatlas.org/data/topic/topic.aspx?cat=18.Accessed 2013 Oct 1.

[8] Periyakoil VS, Noda AM, Kraemer HC (2010) Assessment of factors influencing preservation of dignity at life's end: Creation and the cross-cultural validation of the preservation of Dignity Card-sort Tool. J Palliat Med 13: 495–500. 20420549 10.1089/jpm.2009.0279 PMC2938912

[9] Periyakoil VS, Kraemer HC, Noda A (2009) Creation and the empirical validation of the dignity card-sort tool to assess factors influencing erosion of dignity at life's end. J Palliat Med 12: 1125–1130. 19708793 10.1089/jpm.2009.0123 PMC2939852

[10] Barnato AE, Herndon MB, Anthony DL, Gallagher PM, Skinner JS, et al (2007) Are regional variations in end-of-life care intensity explained by patient preferences? A study of the US Medicare population. Med Care. May 45(5): 386–93. 10.1097/01.mlr.0000255248.79308.41 PMC2147061 17446824

[11] The Dartmouth Atlas of Health Care website. Available: http://www.dartmouthatlas.org/downloads/reports/preference_sensitive.pdf.Accessed 2013 Oct 1.

[12] Gallup GH Jr. Spiritual beliefs and the dying process: A report on a national survey (1997) Conducted for the Nathan Cummings Foundation and the Fetzer Institute.

[13] Blondeau D, Valois P, Keyserlingk EW, Hébert M, Lavoie M (1998) Comparison of patients' and health care professionals' attitudes towards advance directives. J Med Ethics October 24(5): 328–335 PMCID: PMC1377609. 9800589 10.1136/jme.24.5.328 PMC1377609

[14] Hughes DL, Singer PA (1992) Family physician's attitudes toward advance directives. CMAJ 146(11): 1937–1944.). 1596842 PMC1490365

[15] Schüklenk U, Van Delden JJM, Downie J, Mclean S, Upshur R, et al (2011) End-of-Life Decision-Making in Canada: The Report by the Royal Society of Canada Expert Panel on End-of-Life Decision-Making Bioethics. 25(Suppl 1): 1–4. Available: http://www.ncbi.nlm.nih.gov/pmc/articles/PMC3265521/pdf/bioe0025-0001.pdf.Accessed 2013 Oct 1. 10.1111/j.1467-8519.2011.01939.x PMC3265521 22085416

[16] Toller CA, Budge MM (2006) Compliance with and understanding of advance directives among trainee doctors in the United Kingdom. J Palliat Care 22: 141–6. 17058751

[17] Bond CJ, Lowton K (2011) Geriatricians' view on advance decisions and their use in clinical care in England: qualitative study. Age Ageing 40(4): 450–456. 21429949 10.1093/ageing/afr025

[18] Davidson KW, MD; Hackler C, PhD; Caradine DR, MD; McCord RS (1989) MD (1989) Physicians' Attitudes on Advance Directives. JAMA 262(17): 2415–2419 10.1001/jama.1989.03430170077032 2795827

[19] Library of Congress website. S.1766 - Patient Self Determination Act of 1989; Internet reference; accessed on 10–01-2013 Available: http://www.loc.gov/.Accessed 2013 Oct 1.

[20] Kraemer HC, Kupfer DJ (2006) Size of treatment effects and their importance to clinical research and practice. Biol Psychiatry 59(11): 990–6. 16368078 10.1016/j.biopsych.2005.09.014

[21] Hsu LM (2004) Biases of success rate differences shown in binomial effect size displays. Psychol Bull 9: 183–197. 10.1037/1082-989X.9.2.183 15137888

[22] Mann HB, Whitney DR (1947) “On a Test of Whether one of Two Random Variables is Stochastically Larger than the Other”. Annals of Mathematical Statistics,18 (1): 50–60.

[23] Wilcoxon F (1945) “Individual comparisons by ranking methods”. Biometrics Bulletin, 1 (6): 80–83.

[24] Kruskal WH, Wallis WA (1952) “Use of ranks in one-criterion variance analysis”. Journal of the American Statistical Association; 47 (260): 583–621.

[25] Kraemer HC (2008) Sensitivity, Specificity and Receiver Operator Characteristic (ROC) Methods. Wiley Encyclopedia of Clinical Trials. 1–10.

[26] Physician Specialty Data Book; Center of Workforce Studies, Association of the American Medical Colleges Website, (2012) Available https://members.aamc.org/eweb/upload/2012%20Physician%20Specialty%20Data%20Book.pdf Accessed 2013 Oct 1.

[27] Redmann AJ, Brasel KJ, Alexander CG, Schwarze ML (2012) Use of Advance Directives for High-Risk Operations A National Survey of Surgeons. Annals of Surgery Volume 255, Number 3. 10.1097/SLA.0b013e31823b6782 22167006

[28] Schwarze ML, Bradley CT, Brasel KJ (2009) Surgical “buy-in”: The contractual relationship between surgeons and patients that influences decisions regarding life-supporting therapy. Crit Care Med 38: 843–848. 10.1097/CCM.0b013e3181cc466b PMC3042894 20048678

[29] Bradley CT, Brasel KJ, Schwarze ML (2010) Physician attitudes regarding advance directives for high-risk surgical patients: A qualitative analysis. Surgery 148: 209–216. 20580048 10.1016/j.surg.2010.05.020

[30] Cassell J, Buchman TG, Streat S, Stewart RM (2003) Surgeons, intensivists, and the covenant of care: Administrative models and values affecting care at the end of life-Updated. Crit Care Med 31: 1551–1559. 12771632

[31] Gallo JJ, Straton JB, Klag MJ, Meoni LA, Sulmasy DP, et al (2003) Life-Sustaining Treatments: What Do Physicians Want and Do They Express Their Wishes to Others?. Journal of the American Geriatrics Society 51: 961–969 10.1046/j.13652389.2003.51309.x 12834516

[32] Straton JB, Wang NY, Meoni LA, Ford DE, Klag MJ, et al (2004) Physical functioning, depression, and preferences for treatment at the end of life: the Johns Hopkins Precursors Study. J Am Geriatr Soc 52(4): 577–82. 15066074 10.1111/j.1532-5415.2004.52165.x

[33] Christakis NA, Lamont EB (2000) Extent and determinants of error in doctors' prognoses in terminally ill patients: Prospective cohort study. BMJ 320: 469–472. 10678857 10.1136/bmj.320.7233.469 PMC27288

[34] Maltoni M, Caraceni A, Brunelli C, Broeckaert B, Christakis N, et al (2005) Prognostic factors in advanced cancer patients: evidence-based clinical recommendations–a study by the Steering Committee of the European Association for Palliative Care. J Clin Oncol 23(25): 6240–8. 16135490 10.1200/JCO.2005.06.866

[35] Chow E, Harth T, Hruby G, Finkelstein J, Wu J, et al (2001) How accurate are physicians' clinical predictions of survival and the available prognostic tools in estimating survival times in terminally ill cancer patients? A systematic review. Clin Oncol (R Coll Radiol) 13(3): 209–18. 11527298 10.1053/clon.2001.9256

[36] Temel JS, Greer JA, Muzikansky A, Gallagher ER, Admane S, et al (2010) Early palliative care for patients with metastatic non-small-cell lung cancer. N Engl J Med 363(8): 733–42 10.1056/NEJMoa1000678 20818875

[37] Smits HL, Furletti M, Vladeck BC (2002). Palliative care: An opportunity for Medicare. New York: Mount Sinai School of Medicine, Institute for Medicare Practice;

[38] Brotherton SE, Etzel SI (2013) Graduate Medical Education, 2012–2013 JAMA. 310(21): 2328–2346 10.1001/jama.2013.278364 24302103

[39] Wittink MN, Morales KH, Meoni LA, Ford DE, Wang NY, et al. (2008) Stability of preferences for end of life treatment after 3 years of follow-up: The Precursors Study. Archives of Internal Medicine;168(19): :2125–2130. PMCID: PMC2596594. 10.1001/archinte.168.19.2125 PMC2596594 18955642

[40] Blechman JA, Rizk N, Stevens MM, Periyakoil VS (2013) Unmet quality indicators for metastatic cancer patients admitted to intensive care unit in the last two weeks of life. J Palliat Med ((10)) 1285–9 10.1089/jpm.2013.0257. Epub 2013 Sep 10. PMID: 24020919 PMC3791052 24020919

